# Higher Levels of Harsh Parenting During the COVID-19 Lockdown in the Netherlands

**DOI:** 10.1177/10775595211024748

**Published:** 2022-05

**Authors:** Novika Purnama Sari, Marinus H. van IJzendoorn, Pauline Jansen, Marian Bakermans-Kranenburg, Madelon M. E. Riem

**Affiliations:** 1 Department Psychology, Education and Child Studies, Erasmus University Rotterdam, the Netherlands; 2 Generation R Study Group, Erasmus University Medical Center Rotterdam, the Netherlands; 3 Department of Child and Adolescent Psychiatry/Psychology, Erasmus University Medical Centre, Rotterdam, the Netherlands; 4 Clinical Child and Family Studies, Faculty of Behavioral and Movement Sciences, Vrije Universiteit, Amsterdam, the Netherlands; 5 Behavioural Science Institute, Radboud University, Nijmegen, the Netherlands

**Keywords:** child maltreatment, emotional maltreatment, propensity score matching, physical abuse, parenting

## Abstract

Previous studies on the impact of COVID-19 indicate that pandemic-related distress increases risks for child maltreatment, although data on the scope of this problem are still scarce. Here, we assessed whether parents with toddlers (*n* = 206) more often used harsh discipline during the lockdown in the Netherlands compared to a matched parent sample collected prior to the pandemic (*n* = 1,030). Parents were matched on background characteristics using propensity score matching. We found that harsh parenting levels were significantly elevated compared to pre-pandemic levels. Harsh parenting behaviors with a low prevalence before COVID-19 increased most strongly: shaking, calling names, and calling the child stupid. These results suggest that parental tolerance for children’s disobedience is lower under the adverse circumstances of COVID-19 and, as a result, abusive parenting responses are more difficult to inhibit. Thus, a lockdown seems to increase risks for child maltreatment, underscoring the need for effective support strategies for at-risk families.

The COVID-19 pandemic drastically impacted the lives of everyone, including families. Financial insecurity, social isolation, and health concerns resulted in increased psychological distress among parents. Moreover, due to the closures of child care facilities, parents suddenly needed to combine childcare with trying to meet demands from work remotely. These pandemic-related stressors may impede parenting abilities and may increase the risk of using ineffective parenting strategies, such as a harsh disciplinary style. Harsh discipline, defined as parental attempts to control a child using physical punishment (e.g., slapping) or verbal violence (e.g., yelling; Chang et al., 2003), can be harmful for children, even in the case of mild or infrequent harshness (Lansford et al., 2005). More frequent use of severe harsh discipline can even be considered child physical and emotional maltreatment (van IJzendoorn et al., 2020; World Health Organization [WHO], 1999), with long-term negative consequences for children’s development (Gardner et al., 2019).

Previous studies on the impact of COVID-19 on families indicate that pandemic-related distress increases risk for harsh caregiving behaviors. For example, another study showed that mothers with young children who experience worries about social support during the pandemic are less emotionally available for their children and are more likely to lash out to their children (Van den Heuvel et al., 2021). Although the pandemic impacts the lives of all families, parents with toddlers may be particularly vulnerable. During toddlerhood, caregiving load can be high because of increases in parent-child conflict related to the child’s burgeoning autonomy and non-compliance (Crockenberg & Litman, 1990; Klimes-Dougan & Kopp, 1999). Toddlerhood is considered a critical period during which the use of physical disciplinary strategies increases, with parental control strategies shifting to verbal modalities at older ages (Kuczynski et al., 1987). In this study, we therefore examine how the COVID-19 pandemic impacted disciplinary strategies of parents of toddlers.

As pandemic-related distress seems to result in more frequent or more severe use of harsh caregiving behaviors, there are widespread concerns for increased child maltreatment during COVID-19. Indeed, studies show that parents who lost their job or experience financial insecurities, parental anxiety, and depressive symptoms during the pandemic (Coyne et al., 2020) are at risk for mistreating their child (Brown et al., 2020; Lawson et al., 2020). However, data on the scope of this problem are still scarce. A reported prevalence estimate before pandemic COVID-19 indicated that 2.6%–3.7% children in the Netherlands experienced at least one form of child maltreatment (Van Berkel et al., 2020). Remarkably, some papers indicated that reports of child maltreatment have steeply declined during COVID-19 (Martins-Filho et al., 2020; Thomas et al., 2020a), potentially due to school closures and the child’s inability to leave the home. Hence, child maltreatment may be less visible during the pandemic, but not less prevalent. Indeed, many countries affected by COVID-19 indicated increases in reported child maltreatment (WHO, 2020). However, there is a lack of systematic comparisons and the impact of the pandemic on parent-child relationships therefore remains unclear. In the current study, we examined harsh parenting during the COVID-19 lockdown in April–May 2020 in the Netherlands. In order to shed more light on the scope of the impact of COVID-19 on parents and children, we assessed whether parents more often used harsh discipline during the lockdown compared to a matched group of parents prior to the pandemic, using propensity score matching.

## Method

### Participants

#### Participants COVID-19 study

Participants in the current study were specifically recruited for a study on the impact of COVID-19 and then matched with controls from the ongoing population-based Generation R Study (Jaddoe et al., 2007), which collected data on harsh parenting prior to the COVID-19 pandemic. In a dedicated COVID-19 study, 1,156 parents with children aged 1–10 years participated in an online survey on the impact of the pandemic on family life during the COVID lockdown in the Netherlands. Parents were recruited using a snowball sampling strategy, social media advertisements (facebook, linkedin, twitter), and by contacting schools and day care centers. In addition, parents were recruited by distributing the questionnaire among parents who were members of the Dutch I&O research panel (www.ioresearch.nl). Data was collected during the period of closure of schools and day care centers (April 17–May 10, 2020). During the first COVID-19 lockdown in the Netherlands, schools and day care centers were closed between March 16 and May 10, 2020. Data collection thus started 1 month after the lockdown began. At this time, additional governmental pandemic measures included remote working, keeping social distance from others. Dutch people were allowed to leave their home if they had no COVID-19 diagnosis or symptoms and if they had not been exposed to infected others. Permission for the study was obtained from the local ethics committee of the School of Social and Behavioral Sciences of Tilburg University.

#### Participants Generation R

Parents from the COVID-19 sample were compared to a matched sample of parents from the Generation R Study, a prospective population-based cohort study designed to identify determinants of health and development across childhood and adolescence (Jaddoe et al., 2007). The institutional review board of the Erasmus Medical Centre approved the Generation R Study (Jaddoe et al., 2007). Recruitment of parents was done during pregnancy by midwives and obstetricians in 2002–2006. Written informed consent was obtained from all participants. Full consent for the postnatal phase was obtained from 8,305 participants. A questionnaire on harsh parenting was completed by 4,154 families when the children were 3 years old (April 2005–January 2009; see Figure S1).

#### Participant matching procedure

The families from the two samples differed in terms of child age (Generation R: 3 years, COVID-19: 1–10 years range). In order to match the samples, only parents with a child aged 3 years were selected from the COVID-19 sample. Eligible for inclusion were parents of 4,360 children (206 COVID-19, and 4,154 Generation R) of whom 1,236 parents (206 COVID-19, 1,030 Generation R) were selected following matching of the samples (Figure S1). Descriptive statistics of parents and children in the COVID-19 study and in the complete and selected Generation R sample are shown in Table 1. Parental age varied across samples, with parents in Generation R having a lower mean age than parents in the COVID-19 sample [32.6 (± 4.8 *SD*) vs. 35.5 (± 4.3 *SD*) years, respectively]. The majority of children in participating families were Dutch (all four grandparents were born in the Netherlands) both in Generation R (68.4%) and in the COVID-19 sample (97.1%). The percentage of parents with a Dutch ethnicity was lower in the total sample of Generation R compared to the COVID-19 sample, but the groups did not differ in ethnicity after propensity score matching (see Table 1). Most of the parents had a high educational level (higher vocational training and university level) and had a total income of more than 30,000 euros per year. Before propensity score matching, there were statistically significant differences in reporters (mother or father), family income, marital status, child gender, and number of children between the complete Generation R and COVID-19 samples.

**Table 1.** Participant Characteristics.

Characteristics	COVID-19 Sample n = 206	Generation R Matched-Sample n = 1,030	*p* Value^a^	Full Generation R Cohort n = 4,154	*p* Value^b^
Parents					
Reporters (%)					
Mother	79.6	72.7	.227	59.2	.036
Father	20.4	27.3		40.8	
Age (years)	35.5 (4.3)	35.1 (4.4)	.232	32.6 (4.8)	.000
Education (%)					
High	69.9	71.3	.905	66.5	.660
Mid	25.7	24.1		25.4	
Low	4.4	4.7		8.1	
Total Income (%)					
>30,000 euros/year	90.3	89.2	.981	78.8	.051
<30,000 euros/year	9.7	10.8		21.2	
Marital Status (%)					
Married/Living together	96.6	96.0	.700	89.4	.060
No partner	3.4	4.0		10.6	
Number of Children (%)			.681		.000
One child	18.0	16.5		58.9	
Two children	53.9	60.8		30.5	
Three children	20.9	18.4		8.4	
More than four children	7.3	4.3		2.2	
Harsh Parenting Score	2.6 (2.5)	1.9 (1.7)	.000	2.0 (1.9)	.122
*Child*					
Gender (%)			.776		.777
Boy	53.9	52.4		49.7	
Girl	46.1	47.6		50.3	
Ethnicity (%)					
Dutch	97.1	96.8	.999	68.4	.000
Other	2.9	3.2		31.6	

^a^ Difference testing between COVID-19 sample and Generation R matched-sample.

^b^ Difference testing between Generation R matched-sample and Full Generation R cohort.

### Measures

#### Harsh parenting

Six items of the Parent-Child Conflict Tactics Scale (CTSPC; Straus et al., 1998) were selected to constitute a harsh discipline scale (Jansen et al., 2012): “shook my child,” “shouted or screamed angrily at my child,” “called my child names,” “threatened to give a slap, but I didn’t do it,” “angrily pinched my child’s arms,” and “called my child stupid, lazy, or something like that.” This six-item harsh discipline scale was confirmed previously using factor analysis in Generation R (Jansen et al., 2012). In Generation R, the CTSPC was completed by both father and mother. In the COVID-19 sample, harsh parenting was assessed by one of the parents. Parents rated how often they used the different types of disciplinary behavior in the past 2 weeks on a 6-point scale, ranging from never to ≥5 times. Similar to Jansen et al. (2012), categories were combined into three categories “never” (0), “once” (1), and “twice or more” (2), yielding a score ranging from 0 to 12, with higher scores indicating higher severity of harsh discipline. Cronbach’s α was 0.79 in the COVID-19 sample. Previous confirmatory factor analyses indicated good fit for the harsh parenting factor in both mothers and fathers in Generation R (Jansen et al., 2012).

#### Confounders

The following variables were considered potential confounders: child gender and ethnicity; parental age, marital status, education, and income. In Generation R, data on gender and age of the child was obtained from medical records of obstetricians and midwives at birth. The child’s ethnicity was based on the grandparents’ birth countries, and was categorized as “Dutch” and “Other,” both in Generation R and the COVID-19 sample. A questionnaire completed early in pregnancy provided data on parental age and marital status (married/living together, single parenthood). Highest attained educational level of the parent was categorized into: low (no education and primary school only); medium education (secondary school level), and high education (higher vocational training and university level), both in Generation R and the COVID-19 sample. Family income was defined by the total net yearly income of the household (assessed when the children were aged 3 years), and was categorized as < 30,000 euros and >30,000 euros per year.

### Statistical Analyses

All statistical analyses were conducted using R (R Core Team, 2014). In Generation R, we randomly selected one reporter from the mother and father assessments by using the *random* and *ifelse* commands, and we computed parental age at completion of the harsh parenting questionnaire. To compare the samples from COVID-19 and Generation R, we created a matched dataset using propensity score matching. This technique matches COVID-19 study parents to Generation R parents based on measured covariates (Thoemmes, 2012), effectively balancing the covariates (Thomas et al., 2020b). In this study, the measured covariates were reporters (mother or father), child gender and ethnicity, parental age, marital status, number of siblings, parental education, and family income. The analyses were performed by using the package *matchit* (Daniel et al., 2011) with the matching algorithm of nearest neighbor, and the ratio for matching specified to be 1:5. As in previous studies (Mackenbach et al., 2014), harsh discipline scores were square root transformed to achieve a normal distribution. *T* tests were used to examine the mean differences on harsh parenting scores. Additionally, we explored the mean differences for each item using *t* tests.

In addition, we examined whether the number of parents with high harsh parenting scores increased during the COVID lockdown. With a χ^2^ test, we tested whether the number of parents with scores ≥3, which corresponds to the cut-off of 25% highest scores in the population-based Generation R sample prior to the pandemic (Jansen et al., 2012), was significantly higher in the COVID-19 sample compared to Generation R.

## Results

**Figure 1.**
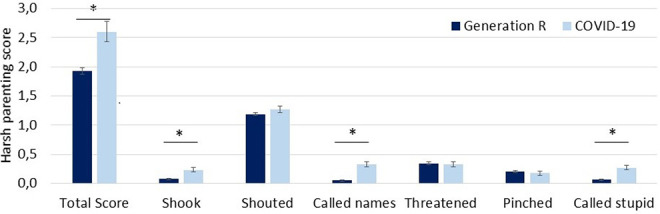
Harsh parenting scores on each item of the CTSPC in the Generation R cohort (*n* = 1,030) and the COVID-19 sample (*n* = 206). *Note.* *p < .001. Error bars represent SE.

The characteristics of the COVID-19 and the Generation R samples before and after propensity score matching are presented in Table 1. Before propensity score matching, the full Generation R cohort and COVID-19 samples differ on reporters (mother or father), parental age, number of children in the household, and child ethnicity. After propensity score matching, the Generation R and COVID-19 samples did not differ on any of the potential confounding variables. Figure 1 indicates that the COVID-19 sample had a higher score on the total harsh parenting scale as compared to the Generation R sample (*t* [1234] = 3.12, *p* < .01, Cohen’s *d* = 0.24). Additionally, t-tests were conducted for each item of the CTSPC. Analyses showed that three specific items determined the difference in the total harsh parenting score. The COVID-19 sample showed higher prevalence of the following items: “shook my child” (*t* [1234] = 5.88, *p* < .001, Cohen’s *d* = 0.45), “called my child names” (*t* [1234] = 10.78, *p* < .001, Cohen’s *d* = 0.82), and “called my child stupid, lazy, or something like that” (*t* [1234] = 8.51, *p* < .001, Cohen’s *d* = 0.65). Effect sizes of the pre- and post- pandemic differences in the item scores were medium to large according to conventional criteria, indicating that the difference is meaningful. The number of parents with scores ≥3 during the COVID-19 lockdown was significantly higher compared to the number of parents with scores ≥3 before the COVID-19 pandemic (Generation R: 25.6%, COVID-19: 34.0%, χ^2^(1, 1236) = 6.1, *p* = .014). See Table 2 for means, standard deviations (before square root transformation) and the (change in) prevalence of the individual items in Generation R and the COVID-19 sample.

**Table 2.** Means, Standard Deviations, and (Change in) Prevalence of the Items of the CTSPC in Generation R and the COVID-19 Sample.

	Generation R					COVID-19					Generation R vs COVID-19	
			Prevalence					Prevalence			Increase in Prevalence	
Item CTSPC	*M*	*SD*	Never	Once	≥ Twice	*M*	*SD*	Never	Once	≥ Twice	Once	≥ Twice
Shook	0.08	0.33	94.0%	4.3%	1.8%	0.23	0.53	81.6%	13.6%	4.9%	+9.3%	+6.7%
Shouted	1.19	0.83	26.4%	28.3%	45.3%	1.27	0.83	24.3%	24.8%	51.0%	−3.5%	+5.7%
Called my child names	0.05	0.28	96.3%	2.2%	1.5%	0.33	0.61	75.2%	17.0%	7.8%	+14.8%	+6.3%
Threatened	0.35	0.66	76.3%	12.8%	10.9%	0.33	0.61	74.3%	18.4%	7.3%	+5.6%	−3.6%
Pinched	0.20	0.51	84.4%	10.2%	5.0%	0.17	0.45	85.4%	11.7%	2.9%	+1.5%	−2.1%
Called my child stupid, lazy, or something like that	0.06	0.27	95.4%	2.8%	1.8%	0.27	0.54	77.7%	17.5%	4.9%	+14.7%	+3.1%

## Discussion

In the current study, we assessed whether Dutch parents with a child aged 3 years used harsh discipline more often during the COVID-19 lockdown compared to a matched sample of parents prior to the pandemic. Using a propensity score matching technique, parents were matched on a range of sociodemographic variables that might confound associations. We found that harsh parenting levels were significantly elevated compared to pre-pandemic harsh parenting. Moreover, the number of parents with high harsh discipline scores increased during the COVID-19 lockdown, indicating that more parents frequently used harsh caregiving responses to discipline their children. Parents were more inclined to shake their child, call their child names, and call their child stupid, lazy, or something like that during the lockdown. These parental behaviors can be considered physical and emotional maltreatment, respectively (van IJzendoorn et al., 2020; WHO, 1999) and may have long-term negative consequences for children’s development.

A remarkable pattern was observed in the analyses with the individual items of the CTSPC. Whereas parents more often shook their child, called their child names, or called their child stupid, no significant increases were observed for parental shouting, threatening to hit the child, and pinching the arm during the lockdown. Interestingly, a previous study conducted prior to the pandemic showed that parents in Generation R less often used shaking, name calling, and calling stupid as disciplinary strategies compared to the other harsh parenting behaviors (Jansen et al., 2012). Hence, harsh parenting behaviors with a low prevalence before COVID-19 seemed to increase most strongly during the lockdown. These behaviors may represent aggressive acts of harsh parenting that are difficult to control under the adverse circumstances of COVID-19. Pandemic-related distress may lower parental tolerance for children’s disobedience and, as a result, may trigger abusive parenting responses that can generally be inhibited by most parents in the absence of adversity.

Historically, child maltreatment is considered one of the serious consequences of pandemics (Peterman et al., 2020). School closures, economic uncertainty, social isolation and disrupted support networks have been mentioned as possible pathways linking pandemic-related disruptions with child maltreatment (Cluver et al., 2020, Peterman et al., 2020). These disruptions increase parental stress, which can trigger harsh parenting. The drastic impact of COVID-19 has even been described as an “evolutionary mismatch” (Dezecache et al., 2020), in which previous coping strategies and patterns of behavior no longer work because of sudden changes in the social environment. This drastic change may particularly hit parents who suddenly experience a collision of roles such as caregiver, teacher, employee or employer, and partner (Coyne et al., 2020). Parents can rely less on their regular support system (e.g., grandparents not being able to take care of the children) or may perceive lack of control over stressful events related to COVID-19, which in turn can increase risk for harsh caregiving or even child abuse (Guterman et al., 2009; Li et al., 2011). Pandemic-proof support strategies, such as dial pad help center or measures reducing caregiving load, are therefore urgently needed for parents during COVID-19.

Some limitations should be noted. First, harsh discipline was measured with a self-report questionnaire that may have resulted in under-report. Second, we focused on parents of 3-year-olds because toddlerhood is considered a critical period during which physical disciplinary strategies increase. It is, however, also important to study how COVID-19 impacts on parents of children in other age categories. Second, in the Generation R sample, the questionnaire on harsh parenting was filled out between 2006 and 2009, thus several years earlier than the Covid-19 sample in 2020. There might be differences between the samples that are explained by time. However, we have statistically matched the samples using propensity score matching, a technique that attempts to reduce the bias due to confounding variables such as changes in SES that could have occurred over time. Moreover, parents’ directive control has decreased rather than increased in the past decades (Trifan, Stattin, & Tilton-Weaver, 2014). Most importantly, in the three waves of the Netherlands’ Prevalence study of Maltreatment (NPM) it has been shown that rates of maltreatment between 2005 and 2017 have remained remarkably stable (Euser et al., 2013; Van Berkel et al., 2020). It is therefore unlikely that the increase in harsh parenting in the COVID-19 sample is explained by time. We must also note that we do not know whether these changes in harsh parenting have resulted in more physical injury or hospitalization. Lastly, a limitation of our study is that the majority of parents who participated in the studies were mothers. In a previous study with partly the same COVID-19 sample, we found that mothers are highly impacted by the pandemic (Guo et al., 2021), perhaps more than fathers because mothers are still often the primary caregiver, spending most time with their child. Future studies with larger sample sizes should include fathers in order to examine whether the pandemic differentially impacts mothers and fathers.

In conclusion, our findings indicate that the COVID-19 pandemic is associated with increases in harsh parenting behaviors and, therefore, may impact the wellbeing of children. In times of pandemics, child abuse may be less visible, yet more prevalent. Hence, with more lockdowns yet to come during COVID-19 or future pandemics, we need to develop effective strategies to strengthen networks of support for at-risk families in order to prevent child maltreatment and its detrimental consequences for children’s development.

## Supplemental Material

Supplemental Material, sj-docx-1-cmx-10.1177_10775595211024748 - Higher Levels of Harsh Parenting During the COVID-19 Lockdown in the Netherlands Click here for additional data file. Supplemental Material, sj-docx-1-cmx-10.1177_10775595211024748 for Higher Levels of Harsh Parenting During the COVID-19 Lockdown in the Netherlands by Novika Purnama Sari, Marinus H. van IJzendoorn, Pauline Jansen, Marian Bakermans-Kranenburg and Madelon M. E. Riem in Child Maltreatment
