# Supplementary material for: Higher Levels of Harsh Parenting During the COVID-19 Lockdown in the Netherlands
Source: Child Maltreat. 2022 May;27(2):156–62. doi: 10.1177/10775595211024748 (PMC9003755; doi:10.1177/10775595211024748)
Supplement: Supplemental Material, sj-docx-1-cmx-10.1177_10775595211024748 - Higher Levels of Harsh Parenting During the COVID-19 Lockdown in the Netherlands [file sj-docx-1-cmx-10.1177_10775595211024748.docx]

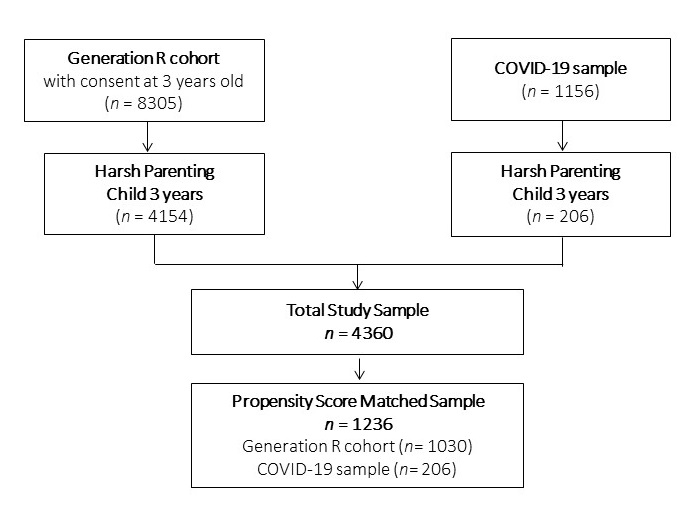


Supplementary Figure 1. Flow chart of participants included for analysis.

Supplementary Figure 1. Flow chart of participants included for analysis.
